# Genetic Diversity and Construction of Salt-Tolerant Core Germplasm in Maize (*Zea mays* L.) Based on Phenotypic Traits and SNP Markers

**DOI:** 10.3390/plants14142182

**Published:** 2025-07-14

**Authors:** Yongfeng Song, Jiahao Wang, Yingwen Ma, Jiaxin Wang, Liangliang Bao, Dequan Sun, Hong Lin, Jinsheng Fan, Yu Zhou, Xing Zeng, Zhenhua Wang, Lin Zhang, Chunxiang Li, Hong Di

**Affiliations:** 1 Key Laboratory of Germplasm Enhancement, Physiology and Ecology of Food Crops in Cold Region, Engineering Technology Research Center of Maize Germplasm Resources Innovation on Cold Land of Heilongjiang Province, Northeast Agricultural University, Harbin 150030, China; song18182746326@163.com (Y.S.); 17332328082@163.com (J.W.); m2335676597@163.com (Y.M.); 15103869531@163.com (J.W.); 15794892115@163.com (L.B.); zhouyu0924@126.com (Y.Z.); zengxing980@hotmail.com (X.Z.); zhenhuawang_2006@163.com (Z.W.); neauzla@163.com (L.Z.); 2 Institute of Forage and Grassland Sciences Heilongjiang Academy of Agricultural Science, Harbin 150086, China; sundequan0451@163.com (D.S.); linhongltt@163.com (H.L.); fanjinsheng0424@163.com (J.F.)

**Keywords:** *Zea mays* L., germplasm conservation, phenotypic evaluation, SNP genotyping, Mahalanobis distance, subgroup classification, seed germination stress

## Abstract

Maize is an essential staple food, and its genetic diversity plays a central role in breeding programs aimed at developing climate-adapted cultivars. Constructing a representative core germplasm set is necessary for the efficient conservation and utilization of maize genetic resources. In this study, we analyzed 588 cultivated maize accessions using agronomic traits such as plant morphology and yield traits such as ear characteristics and single-nucleotide polymorphisms (SNPs) to assess molecular diversity and population structure and to construct a core collection. Nineteen phenotypic traits were evaluated, revealing high genetic diversity and significant correlations among most quantitative traits. The optimal sampling strategy was identified as “Mahalanobis distance + 20% + deviation sampling + flexible method.” Whole-genome genotyping was conducted using the Maize6H-60K liquid phase chip. Population structure analysis, principal component analysis, and cluster analysis divided the 588 accessions into six subgroups. A core collection of 172 accessions was selected based on both phenotypic and genotypic data. These were further evaluated for salt–alkali tolerance during germination, and cluster analysis classified them into five groups. Sixty-five accessions demonstrated salt–alkali tolerance, including 18 with high resistance. This core collection serves as a valuable foundation for germplasm conservation and utilization strategies.

## 1. Introduction

Maize (*Zea mays* L.) is a globally significant crop used for food, feed, and industrial purposes [1]. China ranks third worldwide in saline–alkali soil area, with more than 90% of this land located inland, significantly threatening national food security [2,3]. This is especially critical in northern cold regions, where both low temperatures and severe soil salinization limit crop productivity. Evaluating salt–alkali tolerance in maize germplasm and breeding resistant varieties is therefore essential for improving land use efficiency and maintaining food supply.

Genetic diversity is a cornerstone of maize breeding and improvement. It plays an important role in germplasm utilization and conservation. However, a narrow genetic base remains a limiting factor in breeding progress [4,5]. Germplasm resources are national strategic resources, playing a vital role in sustainable agricultural development [6]. In recent years, with the increasing emphasis on food security in China, research on maize germplasm resources has attracted growing attention. As the basis for germplasm evaluation and utilization, genetic diversity provides critical information for the exploration of gene resources [7].

With advancements in biotechnology, Single-Nucleotide Polymorphisms (SNPs) have become widely used in studies of genetic diversity, QTL mapping, fingerprinting, population structure, and marker-assisted selection [8,9,10]. Core collections aim to reduce redundancy while preserving the genetic diversity of the full germplasm set, facilitating more efficient conservation and utilization [11].

Core collections are typically constructed using either phenotypic data (e.g., agronomic or morphological traits) or molecular data. Several core germplasms of local Chinese maize varieties have been developed based on geographic origin and phenotypic evaluations. Phenotypic diversity assessments of germplasm in China’s National Germplasm Bank have revealed substantial regional variation in diversity indices and traits [12,13,14,15]. Core collections have also been established for crops like wheat, sweet potato, and rice, playing a key role in conserving and using genetic resources [16,17,18,19,20,21]. Previous studies in maize have focused on morphological evaluations and diversity assessments. For example, Cosmos Magorolosho constructed a core collection using phenotypic and genotypic data [22]. The Chinese Academy of Agricultural Sciences (CASA) also developed core germplasms from over 13,000 local varieties and 3000 self-pollinated lines [8].

Other crops have been evaluated for stress tolerance using core collections. For example, sorghum core germplasm has been assessed for salt tolerance during germination [23,24], and soybean core germplasm has been classified into five drought-tolerance levels based on adult-stage assessments [25]. Despite numerous studies on maize genetic diversity and core germplasm development in China, few have focused on germplasm from northern cold regions, and few studies systematically integrated phenotypic and SNP-based assessments of salt tolerance [26,27]. Moreover, few studies integrate both phenotypic and SNP data in constructing specialized salt-tolerant core collections. Heilongjiang Province ranks first in China in terms of maize planting area, production, and commercialization rate. It is also home to the country’s only national medium-term genebank for cold-region crops. With the continuous expansion of germplasm resource collections, challenges have emerged in resource management and utilization. Issues such as insufficient exploration of genetic characteristics and low utilization efficiency have become prominent. Salt–alkali stress, a critical abiotic stress factor in spring maize production areas of northern China, is often accompanied by high pH stress, imposing complex mixed salt–alkali stress on plants and severely impacting food security [28]. Conducting genetic diversity assessment and constructing a core germplasm collection represent effective solutions to bridge the gap between resource conservation and efficient utilization.

In this study, we conducted comprehensive phenotypic and genotypic analyses of a large maize germplasm set. A core collection was established to reduce germplasm redundancy while preserving diversity. Subsequently, salt–alkali tolerance was evaluated during the germination stage to construct a specialized core collection suited for cold-region saline–alkali environments. This curated set will enable more efficient germplasm management and provide a foundation for inbred line development, artificial population construction, and future breeding programs aimed at improving maize stress tolerance.

## 2. Results

### 2.1. Phenotypic and Correlation Analysis

Descriptive statistics and genetic diversity analysis were performed on the 19 phenotypic traits across 588 maize accessions (Table 1). All traits exhibited skewness and kurtosis close to ±1, indicating near-normal distributions and suitability for further statistical analysis (Supplementary Figure S1). The coefficients of variation (CV) ranged from 4.43% to 56.61%, with plot yield exhibiting the highest variability (56.61%) and the growth period showing the lowest (4.43%). Except for seed emergence rate, all traits had CVs above 10%, suggesting substantial variability.

Genetic diversity index (GDI) values ranged from 0.728 to 1.597, with the highest index observed for hundred-grain weight and the lowest for growth period, confirming considerable diversity across the accessions and supporting their value as a diverse germplasm resource.

**Figure 1:**
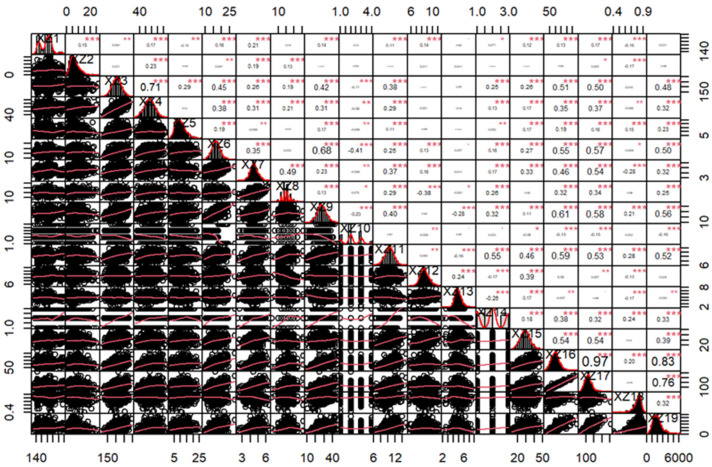
Correlation analysis of the phenotypic traits in maize inbred lines. Note: “*” indicates significance at *p* < 0.05, “** and ***” indicate significance at *p* < 0.01. XZ1-XZ19 represent growth period, number of male spike branches, plant height, ear height, spike stalk length, spike length, spike diameter, spike rows, number of grains in rows, spike type, grain length, grain width, grain thickness, grain type, 100-grain weight, total grain per panicle, dry weight per panicle, seed yield, and plot yield.

Correlation analysis revealed complex interrelationships among traits. Most traits were significantly correlated, though a few showed no association (*p* < 0.05 or *p* < 0.01) (Figure 1). PCA was conducted to reduce dimensionality while retaining critical trait information. Five principal components with eigenvalues greater than 1.0 were extracted, accounting for a cumulative variance contribution of 62.667%, indicating that the majority of the trait variability was captured (Table 2).

### 2.2. Phenotypic Core Collection Construction

Eight core collections were constructed using different systematic clustering methods and evaluated based on trait divergence percentages. The ranking of clustering methods was as follows: Average linkage > flexible method > Ward’s method > complete linkage > centroid method > average linkage > single linkage > unweighted pair-group method using arithmetic averages (Supplementary Table S1). For most traits, the CVs in the core collections were higher than in the original population, indicating preserved or enhanced diversity (Supplementary Table S2).

Evaluation of five core collections, constructed using different sampling proportions (10–30%), showed that each met the criteria of >80% extreme variance compliance and <20% mean difference (Table 3). Based on representativeness, the ranking of sampling proportions was 20% > 15% > 10% > 25% > 30% (Supplementary Table S3).

A total of 24 different sampling strategies were tested by combining two genetic distance types, the top two sampling ratios, two sampling methods, and the two best clustering approaches (Supplementary Table S4). Among them, all 24 strategies had an MD < 20%, with the highest at 5.26%. The VD ranged from 21.05% to 89.47%. The CR ranged from 88.27% to 100%, with eight strategies achieving full (100%) representation. The VR ranged from 108.22% to 124.59%.

The GJ23 strategy, which used Mahalanobis distance, a 20% sampling ratio, deviation sampling, and the flexible method, was identified as the optimal approach for constructing the phenotypic core collection. This strategy achieved >95.00% consistency in capturing unique germplasm. Compared with the original population, the core set demonstrated increased CVs across most traits, reflecting strong heterogeneity (Supplementary Table S5).

**Figure 2:**
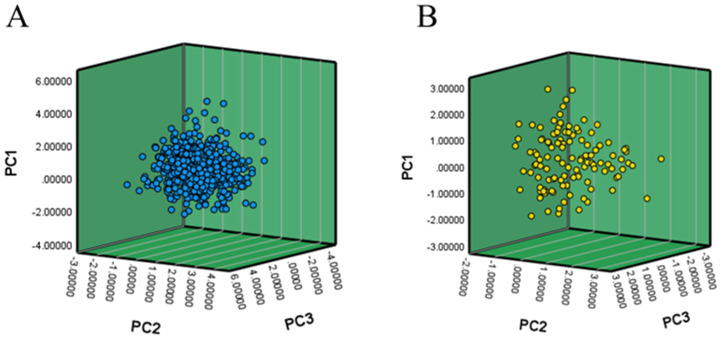
PCA distribution map constructed using phenotypic core germplasm. (**A**) PCA distribution of phenotypic data from the original maize germplasm resources; (**B**) PCA distribution of phenotypic data from the core maize germplasm resources.

PCA comparison between the core and original populations showed that the core collection had a higher cumulative variance (69.76%) compared with the original population (62.67%), suggesting more efficient trait capture and potential reduction in genetic redundancy (Supplementary Table S6). Three-dimensional PCA distribution plots revealed that the original population exhibited dense clustering and high overlap among accessions, indicating genetic similarity among them. In contrast, the core collection showed a more uniform distribution, reduced overlap, and better separation between accessions (Figure 2). These findings confirm that the core collection not only preserves overall diversity but also minimizes genetic duplication.

### 2.3. Genetic Diversity and Population Structure Analysis of Maize Germplasm

**Figure 3:**
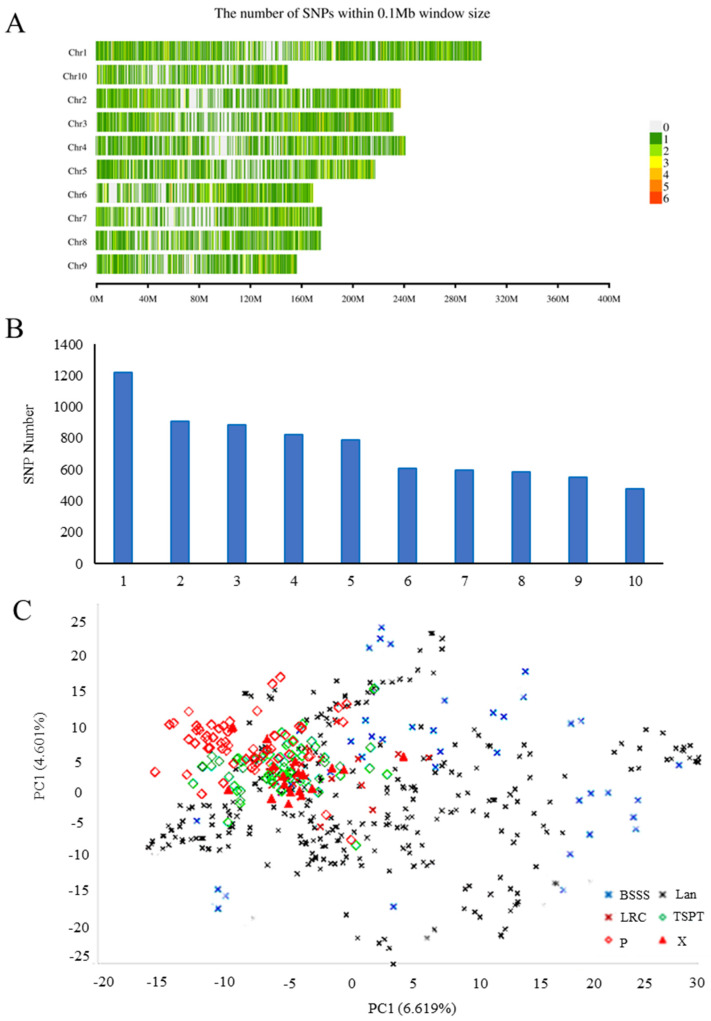
Genomic SNP density of maize germplasms. (**A**) Chromosomal position distribution of genotype data; (**B**) Number of SNPs distributed across each chromosome; (**C**) PCA scatter plot based on SNP genotype data.

A total of 61,214 putative SNPs were identified across the 588 maize accessions. After filtering, 7439 high-quality SNP markers were retained for genetic diversity and population structure analysis. These SNPs were unevenly distributed across the 10 maize chromosomes, with the highest number (1218) located on chromosome 1 and the fewest (476) on chromosome 10 (Figure 3A,B). The SNP density across the genome was higher at the distal ends of the chromosome and lower near the centromeres, indicating reduced diversity and higher sequence conversion near centromeric regions.

PCA principal component analysis showed that the first and second principal components accounted for 6.619% and 4.461% of phenotypic variance, respectively, cumulatively explaining 10.77% (Figure 3C).

**Figure 4:**
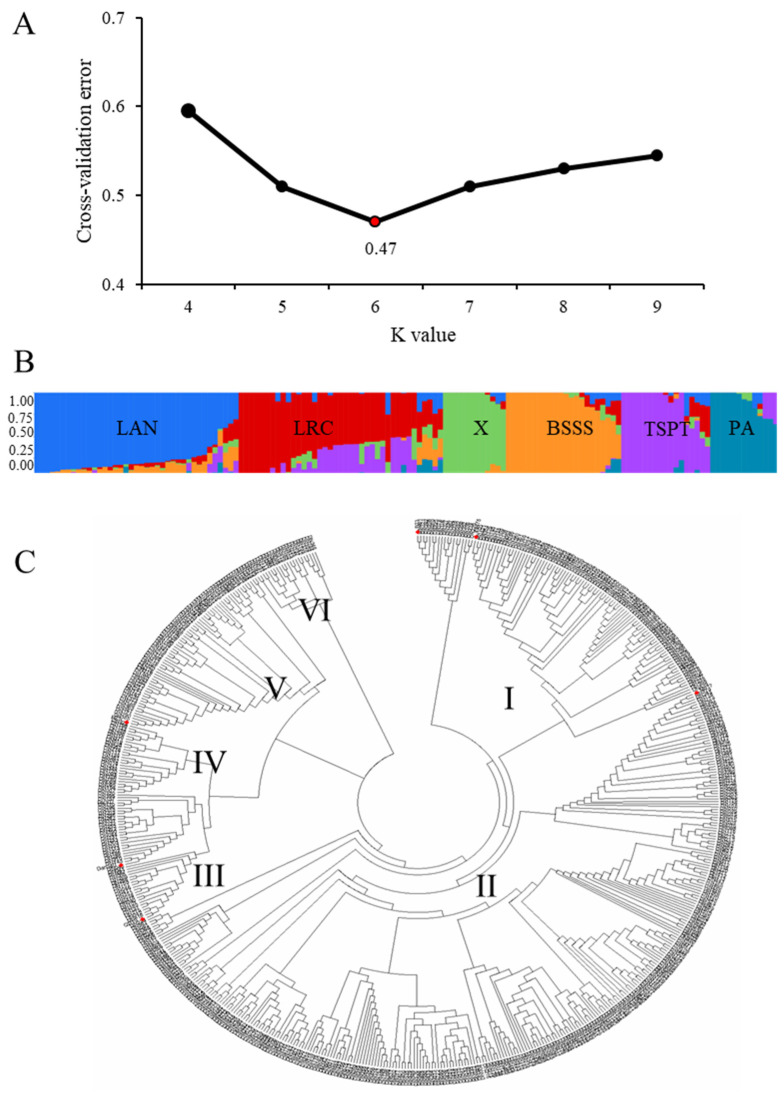
Genetic diversity and population structure analysis of maize germplasm resources. (**A**) Determination of Δ K value; (**B**) Population structure analysis of maize inbred lines; (**C**) Cluster analysis of maize inbred lines.

Population structure analysis was performed using Structure v2.3.4 based on SNP data. The ΔK value peaked at K = 6, indicating that the 588 maize accessions were optimally grouped into six distinct subpopulations (Figure 4A,B). Neighbor-joining cluster analysis also confirmed this grouping pattern (Figure 4C). Among the accessions, the largest group (Branch II) included 376 accessions, while the smallest group (Branch I) contained 19.

The distribution of heterotic groups among these accessions was as follows: Lancaster (Lan), 308 accessions (52.38%); Ludahonggu (LRC), 53 (9.02%); Reid (BSSS), 114 (19.39%); Tangsipingtou (TSPT), 70 (11.90%); P group, 21 (3.57%); and X group, 22 (3.74%) (Figure 4B). Representative inbred lines from each group include B73 and Ye478 (Reid), Dan 340 (LRC), Huangzaosi (TSPT), Qi319 (P), Mo17 (Lan), and Jing 724 (X). These results indicate substantial structure and genetic stratification among northern Chinese maize germplasm, underscoring their breeding potential.

Genetic similarity coefficient (GSC) analysis revealed wide variability, with 50% of accessions falling in the 0.3 < GSC ≤ 0.4 range and the other 50% in the 0.9 < GSC ≥ 1.0 range. The lowest GSC was 0.3098 (between T106 and 196), while the average GSC across all accessions was 0.5595 (Table 4). These results affirm the rich genetic diversity within the studied germplasm population.

### 2.4. Core Collection Construction and Evaluation Based on SNP Markers

Genetic diversity analysis revealed that the observed heterozygosity (Ho) was 0.0316, and the expected heterozygosity (He) was 0.4024, indicating limited heterozygosity within individual accessions (Table 5). These findings suggest that the germplasm exhibits substantial genetic variability, providing a strong foundation for background selection and evolutionary analysis.

Genotypic core collection screening was performed across the 588 maize accessions. Sampling ratios ranging from 10% to 25% captured 99.39% to 99.83% of the total genetic diversity. Diversity indices for these core sets showed Shannon’s information index ranging from 0.5788 to 0.6009 (Table 5).

Based on these genetic diversity results, a 15% sampling ratio was identified as optimal for representing the population’s diversity, followed in rank by 20%, 25%, 30%, and 10%.

**Figure 5:**
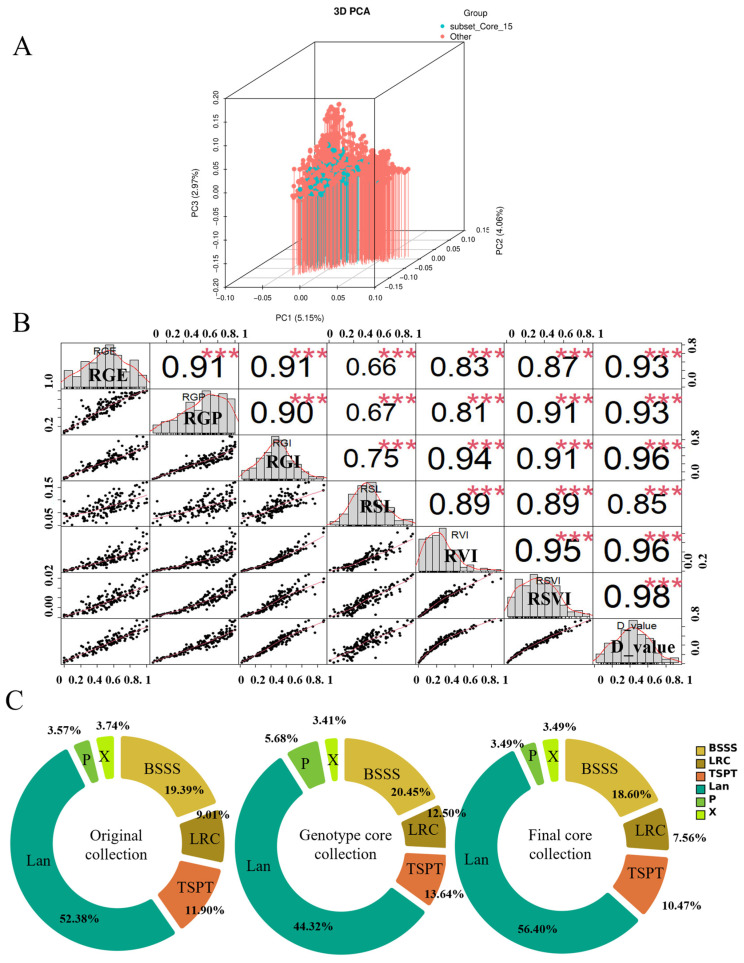
Principal coordinate analysis comparing the core collection with the original population. (**A**) The principal coordinate analysis of core collection and other populations, blue represents the core collection, and red represents the other population. (**B**) The correlation analysis of the phenotypic traits in the core collection. “***” indicate significance at *p* < 0.01.; (**C**) The distribution of heterotic groups of initial population and core collection.

To verify whether the 15% core collection adequately represented the initial population, principal coordinate analysis (PCA) based on genetic distance was conducted using GenAlex 6.5 software. The initial population (blue) showed dense clustering, while the core collection (orange) exhibited a more uniform distribution. This indicates that the 15% core set effectively captured the genetic variability of the full population while reducing redundancy (Figure 5A).

### 2.5. Final Integration of Phenotypic and Genotypic Core Collections

A phenotypic core collection of 117 accessions and a genotypic core collection of 88 accessions were independently constructed. Among them, 33 accessions overlapped between both groups. These 33 shared accessions were integrated to establish a final, non-redundant core collection comprising 172 accessions (Table 6). This combined collection retained comprehensive genetic representation while minimizing duplication, thereby enhancing practical utility.

The final 172 accessions encompassed all six heterotic groups: Lan (97), LRC (13), BSSS (32), TSPT (18), P (6), and X (6) (Figure 5B). The dominance of Lan and the BSSS reflects their prevalence in northern Chinese maize breeding programs.The genetic diversity indices for 172 accessions showed Shannon’s information index is 0.5803, Ho is 0.0367, and He is 0.3957 (Supplementary Table S7). The core collection we finally constructed showed that various genetic diversity indices were highly similar to those of the original germplasm. This core collection serves as a representative subset for future research, breeding, and resource management (Supplementary Table S8).

### 2.6. Identification of Salt–Alkali Tolerant Germplasm in the Core Collection

**Figure 6:**
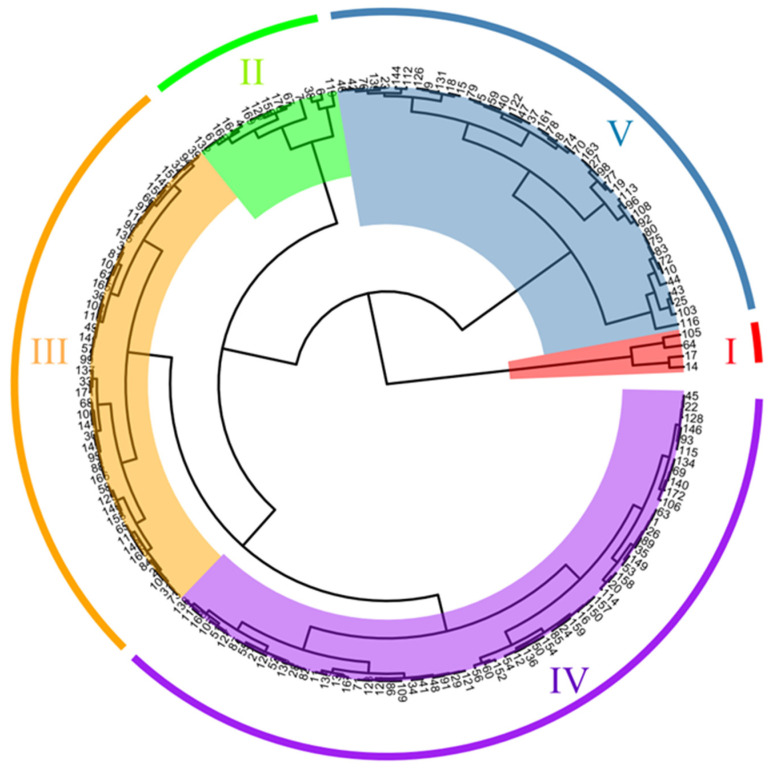
Cluster analysis of core collection accessions for salinity tolerance at the germination stage. Group I: the highly salt–alkaline-tolerant accessions; Group II: the salt–alkaline-tolerant accessions; Group III: the medium salt–alkaline-tolerant accessions; Group IV: the salt–alkaline-sensitive accessions; Group V: the high salt–alkaline-sensitive accessions.

Salt–alkali tolerance was evaluated in all 172 core germplasm accessions during germination. Membership function values were calculated across six physiological indicators. Correlation analysis revealed complex interrelationships among traits. Most traits were significantly correlated, though a few showed no association (*p* < 0.05 or *p* < 0.01) (Figure 5C) Cluster analysis grouped the accessions into five categories based on salt–alkali response (Figure 6, Supplementary Table S7). Group I, comprising four accessions (2.33%), was classified as highly tolerant. Group II included 14 accessions (8.14%) and was categorized as tolerant. Group III, containing 47 accessions (27.33%), exhibited moderate tolerance. Group IV consisted of 64 accessions (37.20%) and was identified as salt–alkali sensitive, while Group V, comprising 43 accessions (25%), was considered highly sensitive to salt–alkali stress.

In total, 65 accessions were classified as salt–alkali tolerant, including 18 that demonstrated strong tolerance. These accessions serve as valuable genetic resources for improving maize performance in saline–alkaline soils and enhancing the utility of marginal lands in northern regions (Supplementary Table S9).

## 3. Discussion

### 3.1. Genetic Diversity Analysis of Maize Germplasm Resources

Crop germplasm resources are strategic assets critical to sustainable agriculture, food security, ecological stability, energy development, and seed industry innovation. The development of new germplasm plays a crucial role in increasing maize yield, enhancing stress resistance, and improving agronomic performance. The processes of germplasm collection, preservation, evaluation, and utilization are foundational to the advancement of high-quality maize breeding programs [29].

A comprehensive understanding of the genetic diversity and variation within breeding populations is crucial for their effective conservation and use [30,31]. Genetic diversity assessment is a fundamental step in breeding research, with morphological variation providing an important dimension of genetic differentiation. In tree species, for instance, broader geographic distribution is often correlated with greater genetic and phenotypic variation, particularly in leaf and physiological traits, underscoring the environmental impact on genetic structure [32].

Studies have consistently demonstrated that landraces exhibit significantly greater genetic diversity compared with elite cultivars [33,34]. Maize, having undergone extensive domestication and selection across diverse regions, has accumulated considerable genetic variation. However, a relatively narrow genetic base continues to constrain modern maize breeding in China. Therefore, expanding and innovating the germplasm base remains a core challenge in maize improvement [35].

Numerous studies have evaluated the genetic diversity and population structure across various maize germplasms, environments, and marker systems. For example, certain landraces contain unexplored genetic variation and selection footprints across regions, much of which remains underutilized in current breeding programs [36,37]. Research on the genetic diversity of 126 inbred lines from the Shaanxi A and Shaanxi B populations revealed six distinct subgroups and low relatedness among lines, reinforcing the genetic richness of regional germplasms [38].

DNA-based molecular markers have proven effective in detecting genetic diversity and in supporting the development of novel cultivars through marker-assisted selection (MAS). This approach not only shortens breeding timelines but also enables targeted selection of parent lines based on their desirable traits. For instance, studies assessing temperate and tropical germplasm have categorized materials based on pedigree, selection history, and endosperm color, providing deeper insight into genetic variation and breeding potential [39,40].

A study on 141 sweet maize inbred lines detected 16,383 high-quality SNP loci, which were grouped into four clusters through PHYLIP analysis, confirming the presence of rich genetic diversity and complex kinship structures [27].

In summary, our findings provide a comprehensive view of the genetic diversity and relationships among 588 maize germplasm resources. This knowledge is valuable for germplasm collection strategies, resource characterization, and breeding innovations in maize.

### 3.2. Population Structure and Genetic Relationships

Analyzing population structure is a crucial first step in genome-wide association studies (GWAS) and in understanding the genetic landscape of breeding populations. Currently, SNP chip technology is widely applied in the division of heterotic groups in maize. Heterosis, defined as the phenomenon where hybrid offspring exhibit superior performance in traits such as growth vigor, yield, and adaptability compared with their parental lines, serves as a fundamental principle in maize breeding. Previous studies have used this technology to classify different types of maize germplasm into four to seven heterotic groups, which provides an important theoretical basis for maize variety improvement and heterosis utilization [41,42]. In our study, structure analysis, PCA, and kinship assessments revealed the presence of six subpopulations within the maize collection, highlighting significant genetic differentiation. Such insights are essential for guiding genetic improvement strategies and understanding evolutionary relationships among accessions.

Genetic differentiation among populations is typically evaluated using fixation indices such as FST [43,44]. Previous studies on 269 widely used temperate maize inbred lines in China, dating back to the 1970s, revealed historical shifts in hybrid utilization patterns and diversity [45]. Similarly, Dube et al. used Evanno’s method to determine an optimal K value of 3, identifying three distinct subgroups [46]. A broader population analysis integrating 237 germplasm samples from seven taxonomic groups and 507 inbred lines produced a high-resolution variation map, offering new insights into maize systematics and genetic diversity [47].

In our PCA, the first and second principal components explained 6.619% and 4.461% of the variance, respectively, accounting for a combined 10.77% of the total variance. STRUCTURE software analysis confirmed K = 6 as the most likely number of subpopulations, consistent with the results of hierarchical clustering. The average GSC among accessions was 0.5595. These findings indicate that the selected maize resources possess unique genetic backgrounds and can serve as a valuable foundation for understanding genetic structure, evolutionary history, and guiding selective breeding efforts.

### 3.3. Linking Genotype and Phenotype in a Newly Constructed Maize Core Collection

While large-scale germplasm collections offer abundant raw material for genetic research and breeding, their sheer size poses challenges in management, evaluation, and practical utilization. To address this, the concept of a “core collection” was introduced, providing a targeted strategy to reduce redundancy while preserving genetic diversity [48,49,50,51].

Since its inception, the core collection framework has been extensively studied worldwide, with efforts focused on construction methodologies, sampling strategies, and analytical techniques to ensure representativeness. Recent advancements in molecular marker technologies, such as SSRs (Simple Sequence Repeats), SNPs, and resequencing, have significantly enhanced the accuracy of core collection assembly by enabling precise assessments of genetic diversity [52,53,54,55]. This approach has already been successfully applied in multiple plant species [56,57,58]. Well-constructed core collections can capture more than 90% of a germplasm bank’s allelic diversity while representing just 10–20% of its accessions [59,60,61,62].

In our study, we integrated phenotypic diversity, genotypic variation, and trait associations to construct a comprehensive maize core collection. Ultimately, 172 accessions (29.25% of the total) were selected, capturing a broad genetic base. Salt–alkali tolerance was evaluated for all 172 core germplasm accessions during germination: 65 accessions were classified as salt–alkali tolerant, including 18 with strong tolerance. These accessions serve as invaluable genetic resources for improving maize performance in saline–alkaline soils, particularly for enhancing the utilization of marginal lands in northern regions. They also represent prime breeding material for gene function studies, marker-assisted selection (MAS), and new variety development. The establishment of this unified core collection provides a practical model for balancing genetic diversity with efficient germplasm use in maize breeding, offering both theoretical insights into genetic conservation and tangible applications for crop improvement.

## 4. Materials and Methods

### 4.1. Plant Materials

**Figure 7:**
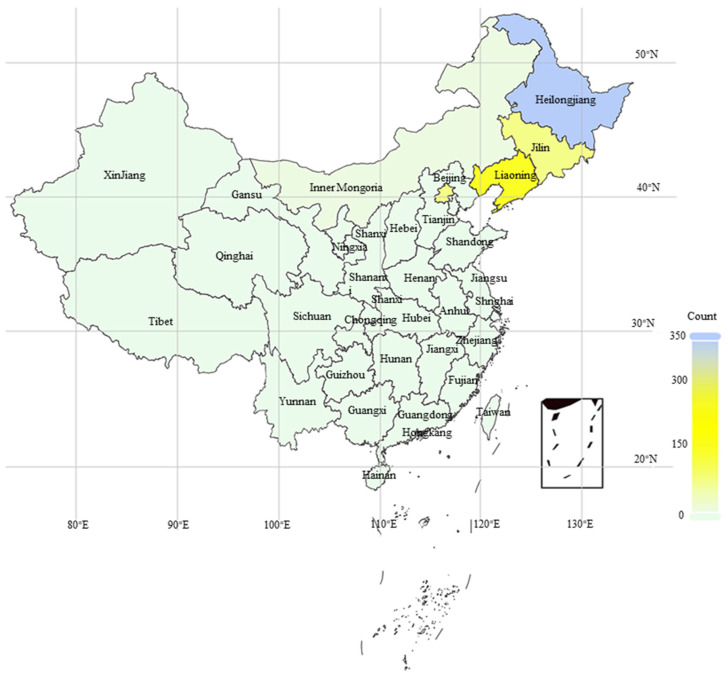
Distribution of maize germplasm resources across China.

This study involved 588 maize (*Zea mays* L.) inbred lines planted in Heilongjiang Province, China, during the 2023 and 2024 growing seasons. Field traits were assessed from August to September each year. The accessions were collected from diverse sources, including 148 lines from northern maize breeding institutions, 36 European germplasm resources, 34 self-developed inbred lines, and 370 parental inbred lines of commercially important hybrids from eastern and northern China (Figure 7) (Supplementary Table S10).

### 4.2. Phenotypic and Physiological Trait Assessment

All materials were planted at the experimental base of Northeast Agricultural University, located in Xiangyang Township, Xiangfang District, Harbin. Plots consisted of three rows per line, with each row measuring 5 m. For each inbred line, morphological data were collected from 10 replicate plants in each experimental plot. The field trials were conducted in two replications, following standard agronomic practices. Three plants were randomly selected from the middle row of each plot to assess phenological stages. According to the standard process for maize phenotype identification, 19 indicators, including plant architecture, ear morphology, grain characteristics, and yield-related traits, totaling 19 phenotypic indicators [46]. These traits were quantitatively evaluated across both growing seasons.

Trait means were tested for normal distribution and visualized through distribution plots. Correlation analyses among traits were conducted using R 4.3.1. A weighted affiliation function was used to evaluate the 19 traits across all accessions. Microsoft Excel 2016 was used to record data and compute mean values. Trait standardization was performed using Z-scores in SPSS v20.0 to ensure comparability across traits. One-way analysis of variance (ANOVA) and Duncan’s new multiple range test were performed in SPSS v20.0 (IBM, Armonk, NY, USA), with statistical significance set at *p* < 0.05. All results are presented as mean ± standard deviation (SD), based on three biological replicates.

Data visualizations were generated using Origin Pro 2019 (OriginLab Corporation, Northampton, MA, USA), RStudio v3.6.1, and GraphPad Prism 8.0.0 to ensure clarity and reproducibility.

### 4.3. Construction of Core Collection Based on Phenotypic Data

Core collection construction based on phenotypic traits was conducted using QGA Station 2.0. Euclidean distance was selected as the similarity metric, with an initial sampling ratio of 10% applied. Random sampling was combined with eight distinct systematic clustering approaches, including shortest distance, longest distance, intermediate distance, centroid, class averaging, variable class averaging, flexible method, and sum-of-squares of deviations. Methods were evaluated based on their ability to represent the full diversity of the original population [63,64,65]. Subsequently, core collections were generated using Euclidean distance with five different sampling ratios (10%, 15%, 20%, 25%, and 30%). These were assessed using different combinations of sampling methods and clustering algorithms [55]. The best-performing methods were selected based on a combination of statistical metrics. The mean difference (MD) was less than 20%, with smaller values indicating better performance. The coincidence rate of range difference (CR), variance difference (VD), and coefficient of variation (VR) had greater genetic representativeness, the higher their value.

Student’s *t*-tests and F-tests were used to compare the core collection with the original population in terms of trait means and variances. A higher variance, greater coefficient of variation, and minimal mean differences were taken as indicators of an optimally representative core set.

### 4.4. DNA Extraction and SNP Genotyping

Ten seeds were selected from each germplasm and planted in the seedling tray. Genomic DNA was extracted from fresh leaf tissue using a plant genomic DNA extraction kit (Tiangen, Beijing, China). DNA quality and concentration were confirmed by 1% agarose gel electrophoresis [20].

Genotyping of the 588 maize inbred lines was performed using the Maize6H-60K chip. Genotyping was outsourced to Heilongjiang Kenfeng Agricultural Technology Co., Ltd. (Harbin, China). A total of 61,214 SNP loci were initially identified. Using TASSEL 5.0 software, these were filtered based on a minimum allelic variation frequency (MAF) > 0.01 and missing data proportion (PMS) < 0.01, yielding 7439 high-quality SNPs for downstream analysis. These thresholds were chosen to ensure that rare alleles and loci of poor quality were excluded according to previous criteria.

### 4.5. Genetic Diversity Analysis

Genotypic data were analyzed, and genetic distances among maize germplasm resources were estimated using TASSEL software [66]. The expected heterozygosity (He) and observed heterozygosity (Ho) estimate the probability of heterozygosity at a given locus. Lastly, Shannon’s Information Index (I) quantified overall genetic diversity.

All calculations were performed using GenAlEx v6.501 software [67]. Additional parameters, including gene diversity (D) and allele number (N), were analyzed using Power Marker v3.25 [68].

Cluster analysis was performed using the Neighbor-Joining method, and phylogenetic trees were constructed in MEGA 7. A genetic distance heatmap and pairwise similarity matrix were generated using R, which also calculated SNP density and generated corresponding visualizations [26,69].

### 4.6. Population Structure and PCA

Population structure was inferred using a Bayesian model-based clustering algorithm in STRUCTURE v2.3.4 [70]. The admixture model was applied with a burn-in period of 100,000 iterations followed by 100,000 Markov Chain Monte Carlo (MCMC) replications. Values of K (the assumed number of subpopulations) ranging from 1 to 10 were tested across three independent runs. The optimal number of subpopulations was determined using the ΔK method via STRUCTURE HARVESTER [71,72].

PCA was conducted using TASSEL v5.0 based on the 7439 high-quality SNP markers [66].

### 4.7. Core Collection Construction

To develop the core collection, the genetic distance matrix was input into Core-Hunter 3. Sampling was conducted at 16 different proportions (5%, 10%, 15%, 20%, 25%, 30%, 35%, 40%, 45%, 50%, 55%, 60%, 65%, 70%, 75%, and 80%) to control sampling density and assess representativeness [73,74].

The final core collection was established by combining selections based on both genotypic and phenotypic data. Accessions selected solely by phenotypic analysis but not present in the molecular-based subset were added to ensure full representation. Germplasms not included in the final selection were designated as the reserve set [55].

### 4.8. Evaluation of Salinity Tolerance in the Core Collection

To evaluate salt–alkali tolerance, seeds from the core collection were soaked for six hours in a mixed saline–alkaline solution containing 150 mmol/L of sodium ions. The solution was prepared using a 1:9:9:1 ratio of NaCl, Na_2_SO_4_, NaHCO_3_, and Na_2_CO_3_.

Post-soaking, seeds were transferred to filter paper within culture dishes for germination. Germination potential was assessed on Day 4, while germination rate and seedling length were measured on Day 7 [75].

## 5. Conclusions

Population structure analysis, principal component analysis, and cluster analysis divided 588 accessions into six subgroups. A core collection of 172 accessions was selected based on both phenotypic and genotypic data. Among them, 65 accessions were identified as salt–alkali tolerant, including 18 with strong tolerance during the germination stage.

## Figures and Tables

**Table 1:** Statistical analysis and genetic diversity index of 19 phenotypic traits.

Trait	Average Value	Median	Variance	Skewness	Kurtosis	Minimum	Maximum	Average Value	Standard Deviation	Coefficient	Genetic Diversity Index
growth period	148	153	43.183	−1.59	2.032	34	123	148.47	6.571	4.43%	0.728
tassel branch number	7	6	18.61	0.993	1.424	27	0	7.78	4.314	55.45%	1.204
plant height	203.3572	212.33	821.345	−0.083	−0.094	166.66	110.67	203.36	28.659	14.09%	1.556
ear position height	76.2162	75	390.371	0.084	−0.38	106.67	25.33	76.22	19.758	25.92%	1.673
ear stem length	8.946	8	14.68	0.565	0.852	23	0	8.95	3.832	42.82%	0.812
ear length	16.5663	17.6	8.616	0.101	0.563	23.2	5.43	16.57	2.935	17.71%	1.005
ear thickness	4.5824	4.6	0.265	−0.051	1.287	3.98	2.42	4.58	0.515	11.24%	1.566
ear row number	14	14	6.417	0.546	0.795	16	10	14.8	2.533	17.11%	1.41
row grain number	27	27	35.864	0.236	0.237	37	9	27.05	5.989	22.14%	0.881
ear type	2	2	0.692	0.281	−0.574	3	1	2.12	0.832	39.25%	1.205
grain length	10.243	10.48	1.664	−0.146	0.524	8.83	6.27	10.24	1.29	12.60%	1.519
grain width	8.6308	8.7	0.88	0.104	0.389	5.94	5.49	8.63	0.938	10.87%	1.283
grain thickness	4.9404	5	0.399	0.487	1.196	5.5	2.5	4.94	0.632	12.79%	0.827
grain type	2	2	0.538	0.262	−1.109	2	1	1.84	0.733	39.84%	1.052
hundred grain weight	28.5971	32.73	33.585	0.047	0.323	37.87	12.1	28.6	5.795	20.26%	1.597
total grain per ear	99.1884	117.33	1620.006	0.813	1.88	275.5	12	99.19	40.249	40.58%	1.441
dry weight per ear	125.4884	140.33	2542.562	0.886	2.683	397.67	19	125.49	50.424	40.18%	1.4
seed emergence rate	0.7922	0.84	0.006	−1.597	4.559	0.53	0.4	0.79	0.074	9.37%	1.059
plot yield	1546.9988	1056	766819.916	1.136	1.632	5310.99	148	1547	875.683	56.61%	1.178

**Table 2:** Principal component analysis of phenotypic traits.

Indices	Component 1	Component 2	Component 3	Component 4	Component 5
growth period	0.166	0.285	0.241	−0.033	−0.682
tassel branch number	0.069	0.191	0.492	−0.267	0.283
plant height	0.703	0.034	0.169	−0.328	0.155
ear position height	0.513	0.075	0.35	−0.4	0.51
ear stem length	0.292	0.048	−0.252	−0.197	−0.052
ear length	0.689	0.185	−0.189	−0.422	−0.074
ear thickness	0.674	0.254	0.416	0.22	−0.074
ear row number	0.44	−0.291	0.596	0.225	−0.152
row grain number	0.678	−0.252	−0.218	−0.367	−0.087
ear type	−0.249	−0.219	0.355	0.458	0.185
grain length	0.639	−0.12	−0.003	0.4	0.223
grain width	0.013	0.702	−0.233	0.168	0.193
grain thickness	−0.107	0.614	−0.03	0.184	−0.058
grain type	0.406	−0.414	0.095	0.25	−0.019
hundred grain weight	0.441	0.461	−0.287	0.427	0.272
total grain per ear	0.871	−0.071	−0.18	0.177	0.029
dry weight per ear	0.873	0.059	−0.074	0.163	−0.03
seed emergence rate	0.024	−0.554	−0.486	0.099	0.233
plot yield	0.747	−0.094	−0.204	0.13	−0.345
Eigenvalue	5.377	2.015	1.715	1.552	1.248
Contribution (%)	28.302	10.604	9.029	8.167	6.566
Cumulative contribution	28.302	38.906	47.935	56.101	62.667

**Table 3:** Percentage difference between the core collections and the initial population constructed using different sampling proportions.

Construction Proportion	Mean Difference Percentage	Percentage of Variance Difference	Coincidence Rate of Range	Variable Rate of Coefficient of Variation
10%	5.26	78.95	91.92	127.74
15%	5.26	78.95	93.71	121.3
20%	5.26	78.95	96.49	118.28
25%	5.26	73.68	96.92	116.34
30%	10.53	73.68	96.92	114.93

**Table 4:** Germplasm pairs with the largest and smallest genetic similarity coefficients.

Germplasm 1	Germplasm 2	GSC	Germplasm 1	Germplasm 2	GSC
T106	196	0.3366	Dan6263	C260	1.0000
D5801	Jiuyi115	0.3368	M60	P2237	1.0000
zheng58	Jiuyi115	0.337	A22	Jiang134	1.0000
5311	Jiuyi115	0.3372	4112	5022 (B)	1.0000
Qing795	Jiuyi115	0.3374	4112	Ji4112	1.0000
Jing388	Jiuyi115	0.34	5022 (B)	Ji4112	1.0000
Ming84	Jiuyi115	0.3408	4112	N528-1 (1284)	1.0000
T106	KWCB1	0.342	5022 (B)	N528-1 (1284)	1.0000
T106	Yuanfuhuang	0.3424	Ji4112	N528-1 (1284)	1.0000
P138	Jiuyi115	0.3425	Mo113	M0113	1

Note: GSC = Genetic similarity coefficient.

**Table 5:** Comparison of genetic parameters between the core collection and the initial population under different sampling proportions.

Subset (%)	Number of Cultivars	Shannon’s Information Index	Correct_Shannon’s Information Index	Ho	He
Core_5	48	0.6009	0.1552	0.0153	0.4142
Core_10	96	0.5788	0.1268	0.0332	0.3953
Core_15	145	0.5887	0.1183	0.028	0.4036
Core_20	193	0.5844	0.1111	0.038	0.4001
Core_25	242	0.5867	0.1069	0.0362	0.4019
Core_30	290	0.5875	0.1036	0.0351	0.4025
Core_35	338	0.5872	0.1008	0.0291	0.4024
Core_40	387	0.5848	0.0981	0.0314	0.4004
Core_45	434	0.5934	0.0977	0.0325	0.4075
Core_50	483	0.5862	0.0948	0.0287	0.4015
Core_55	532	0.5857	0.0933	0.0361	0.401
Core_60	580	0.585	0.0919	0.0324	0.4005
Core_65	628	0.5892	0.0914	0.0312	0.404
Core_70	677	0.5856	0.0899	0.0318	0.401
Core_75	724	0.5871	0.0891	0.0342	0.4022
Core_80	773	0.5862	0.0881	0.032	0.4015
Core_100	588	0.5868	0.0853	0.0321	0.402

Note: Ho = observed heterozygosity; He = expected heterozygosity.

**Table 6:** Number of phenotypic and genotypic accessions included in the core collection.

Final Core Collection		Phenotypic Traits		SNP Marker	
Reserved Number	Percentage (%)	Reserved Number	Percentage (%)	Reserved Number	Percentage (%)
172	29.25	117	19.90	88	14.97

## Data Availability

The data presented in this study are available upon request from the corresponding author.

## Supplementary Materials

The following supporting information can be downloaded at: https://www.mdpi.com/article/10.3390/plants14142182/s1, Supplementary Figure S1: Frequency distribution of 19 phenotypic traits. Supplementary Table S1: Percentage differences between core collections and the initial population constructed using different clustering methods. Supplementary Table S2: Differences between core collections and the initial population constructed using different sampling proportions. Supplementary Table S3: Summary of core collection construction methods. Supplementary Table S4: Percentage differences between core collections and the initial population constructed using different construction methods. Supplementary Table S5: Comparison of differences between core collections and the initial population constructed using different construction methods. Supplementary Table S6: Principal component analysis between the core collections and the initial collections. Supplementary Table S7: The genetic diversity indices between 172 core collection and initial collection. Supplementary Table S8: Comprehensive evaluation and ranking of 172 maize core germplasm accessions for salinity tolerance at the germination stage. Supplementary Table S9: Strong salinity-tolerant accessions identified from the core germplasm collection during the germination-stage screening. Supplementary Table S10: Names of 588 Chinese maize germplasm resources and their heterosis group classification.
