# Supplementary figures and images for: Genetic Diversity and Construction of Salt-Tolerant Core Germplasm in Maize (Zea mays L.) Based on Phenotypic Traits and SNP Markers
Source: Plants (Basel). 2025 Jul 14;14(14):2182. doi: 10.3390/plants14142182 (PMC12298868; doi:10.3390/plants14142182)

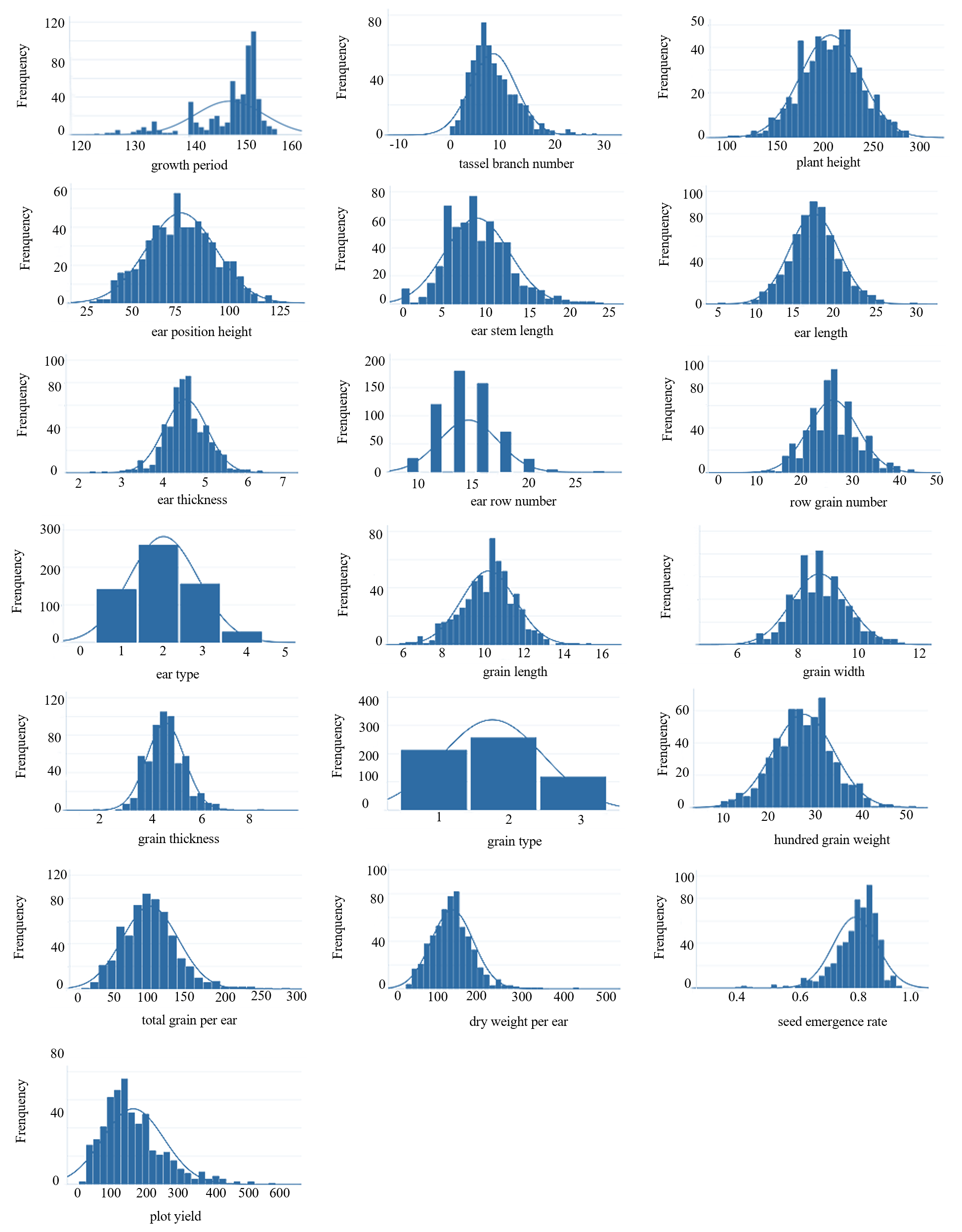

Supplement: Supplementary file 1 [file plants-14-02182-s001.zip › Supplementary Figure S1.tif]
